# The comparative landscape of duplications in *Heliconius melpomene* and *Heliconius cydno*

**DOI:** 10.1038/hdy.2016.107

**Published:** 2016-12-07

**Authors:** A Pinharanda, S H Martin, S L Barker, J W Davey, C D Jiggins

**Affiliations:** 1 Department of Zoology, University of Cambridge, Cambridge, UK

## Abstract

Gene duplications can facilitate adaptation and may lead to interpopulation divergence, causing reproductive isolation. We used whole-genome resequencing data from 34 butterflies to detect duplications in two *Heliconius* species, *Heliconius cydno* and *Heliconius melpomene*. Taking advantage of three distinctive signals of duplication in short-read sequencing data, we identified 744 duplicated loci in *H. cydno* and *H. melpomene* and evaluated the accuracy of our approach using single-molecule sequencing. We have found that duplications overlap genes significantly less than expected at random in *H. melpomene*, consistent with the action of background selection against duplicates in functional regions of the genome. Duplicate loci that are highly differentiated between *H. melpomene* and *H. cydno* map to four different chromosomes. Four duplications were identified with a strong signal of divergent selection, including an odorant binding protein and another in close proximity with a known wing colour pattern locus that differs between the two species.

## Introduction

Gene duplications occur frequently in eukaryotic genomes, where duplication rates are on the order of 0.01 per gene per million years (Lynch and Conery, 2000). Duplication is considered to be the main mechanism by which new genes arise (Katju, 2012), providing material for the origin of evolutionary novelties (Hunt *et al.*, 1998; Manzanares *et al.*, 2000; Kassahn *et al.*, 2009; Qian and Zhang, 2014). For example, the frequency of gene copy-number variants (CNVs) increased during experimental evolution experiments in *Caenorhabditis elegans* (Farslow *et al.*, 2015) and, in *Escherichia coli*, a tandem gene duplication was responsible for the evolutionary novelty in citrate metabolism seen in the long-term evolution experiment (Blount *et al.*, 2012). Such variation shapes gene expression profiles and influences phenotypic diversity (Feuk *et al.*, 2006; Iskow *et al.*, 2012; Katju and Bergthorsson, 2013).

The most common outcome for gene duplicates is to become pseudogenes through the accumulation of deleterious mutations (Lynch and Conery, 2000). Preservation of duplicate genes by natural selection may depend on whether or not one of the two gene copies accumulates mutations that lead to novel beneficial functions (Ohno, 1970). For example, trichromatic vision in Old World primates evolved by duplication of an X-linked opsin gene, an example of *neofunctionalization* (Hunt *et al.*, 1998). In addition, preservation of gene duplicates by natural selection may also occur by selection for increasing gene dosage as shown for ancient duplicates of *Saccharomyces cerevisiae* (Conant and Wolfe, 2008) or for regulatory robustness (Keane *et al.*, 2014). The duplication event does not, however, need to span the complete length of the gene. For example, a partial gene duplication is responsible for the origin of the antifreeze glycoprotein in Antarctic fish (Deng *et al.*, 2010). Alternatively, in *subfunctionalization* models, duplicates are preserved through each copy adopting a subset of the functions of the ancestral gene (Lynch and Force, 2000). This might occur when, for example, regulatory elements of the duplicate loci accumulate mutations that enable both duplicates to take on new functions different to that of the ancestral gene. In zebrafish, *engrailed-1* and -*1b* are a duplicate pair of transcription factors that evolved complementary expression patterns (Force *et al.*, 1999).

Gene duplication can also contribute to speciation. Duplicate genes can provide the raw material for populations to evolve divergent strategies and adapt to novel habitats, or may lead to genetic incompatibilities (Ting *et al.*, 2004). As such, diversification in gene function between duplicated genes can potentially contribute to reproductive isolation. In *Arabidopsis thaliana* recessive embryo lethality is explained by the divergent evolution of two paralogues of a duplicate gene important for the catalyses of the biosynthetic pathway producing histidine. The reciprocal gene loss has led to genetic incompatibilities in specific crosses (Bikard *et al.*, 2009).

Historically, CNVs were identified with cytogenetic technologies such as fluorescence *in situ* hybridization and karyotyping. More recently, array-based comparative genomic hybridization and single-nucleotide polymorphism array approaches have been used. However, array experiments have several weaknesses including limited coverage of the genome, hybridization noise and difficulty in detecting novel and rare variants (Zhao *et al.*, 2013). It is now possible to detect CNVs using next-generation sequencing technology that generates millions of randomly sampled short (100–300 bp) reads in a single run. Several methods have been developed to detect CNVs from short-read data: (1) analysis of abnormally mapping read pairs (paired-end (PE)); (2) analysis of the number of reads aligned to regions of the genome, or read depth (RD); (3) analysis of clipped/gapped alignments, or split reads (SRs); and (4) *de novo* assembly of resequenced genomes (Ye *et al.*, 2009; Abyzov *et al.*, 2011; Rausch *et al.*, 2012; Chen *et al.*, 2014). In order to increase the accuracy and confidence of the calls, a common approach is to integrate the different strategies into a pipeline where complementary signals are incorporated (Mills *et al.*, 2011; Lin *et al.*, 2015; Tattini *et al.*, 2015; Teo *et al.*, 2012). CNVs have now been surveyed across the genomes of a range of closely related species or populations such as sticklebacks, pea-aphids, pigs and fruit-flies (Chain *et al*, 2014; Feulner *et al.*, 2013; Duvaux *et al.*, 2015; Paudel *et al.*, 2015; Rogers *et al.*, 2015).

Here we investigate duplications in the genomes of two species of Neotropical *Heliconius* butterfly. This taxonomic group has been studied for over 150 years since the first evolutionists became fascinated with their striking wing pattern diversity. Since then, *Heliconius* has contributed to answering evolutionary questions covering a broad range of research topics from taxonomy to ecology, behaviour and genetics (Merrill *et al.*, 2015). The best studied species pair are *Heliconius cydno* and *Heliconius melpomene*, two hybridizing sympatric species that differ in their ecology, mimicry patterns and mate preferences. They show low levels of inter-specific hybridization that nonetheless results in genome-wide signatures of admixture (Martin *et al.*, 2013). An outstanding question remains over the number and identity of the genomic regions that contribute to their speciation.

Genetic studies of *Heliconius* butterflies have focussed on loci controlling colour patterns, with many races diverging at these loci alone (Nadeau *et al.*, 2011; Martin *et al.*, 2013). Strong and rapid ecological divergence seems to be a driver of the earliest stages of speciation (Jiggins *et al.*, 2001; McMillan *et al.*, 1997; Muñoz *et al.*, 2010). However, recently, gene duplication in the genus has been linked to the evolution of visual complexity, development and immunity (The Heliconius Genome Consortium, 2012), as well as female oviposition behaviour (Briscoe *et al.*, 2013). Moreover, Nadeau *et al.* (2011) identified multiple CNVs between different *Heliconius* races. These results make *Heliconius* butterflies a promising system for an investigation of evolution by gene duplication for both autosomal and sex-linked genes.

We identify duplications using PE, SR and RD information from whole-genome resequencing short-read data for two *Heliconius* species, *H. cydno* and *H. melpomene*, using a similar strategy to the one used to discover and genotype structural variants in the human 1000 Genomes Project (Mills *et al.*, 2011) and the *Drosophila melanogaster* Genetic Reference Panel (Zichner *et al.*, 2013). By integrating different variant calling algorithms, and taking advantage of three distinctive next-generation sequencing signals, we map duplications among wild-caught *Heliconius* samples from two different species and three different locations, and identify loci putatively under divergent selection that may play a role in speciation.

## Materials and methods

### DNA sequence data retrieval and mapping of short-read data

Illumina (San Diego, CA, USA) paired-end sequencing data for 20 *H. melpomene* and 14 *H. cydno* butterflies (SRA106228, Kronforst *et al.*, 2013; ERP002440, Martin *et al.*, 2013) was downloaded from public repositories using the NCBI SRA toolkit (v2.5.7; National Center for Biotechnology Information, Bethesda, MD, USA). The reads were aligned to the *H. melpomene* genome (v2.0) (Davey *et al.*, 2016) with Stampy (v1.0.23; Lunter and Goodson, 2011) using default values for all parameters except the substitution rate, which was set to 0.01. Picard (v1.128) (picard.sorceforge.net) was used to convert SAM/BAM files and remove PCR duplicate read pairs. Bcftools (v1.3; Li *et al.*, 2009) and bedtools (v2.20.1-13-g9249816; Quinlan and Hall, 2010) were used to process BAM and VCF files (Supplementary Table S1).

### Detecting duplications through the analysis of SR, PE and RD information

The structural variant discovery methods DELLY (v0.6.1) (Rausch *et al.*, 2012), CNVnator (v0.3.2) (Abyzov *et al.*, 2011) and Pindel (v0.2.5a7) (Ye *et al.*, 2009) were used to detect candidate duplications in a focal set of 10 *H*eliconius *melpomene rosina* and 10 *Heliconius cydno galanthus* from Costa Rica, representing the largest population sample available for each species. We ran DELLY and Pindel on each population and CNVnator on each sample individually. These algorithms analyse different sequence signals to call the putative duplications: DELLY uses SR and PE information, Pindel uses SR information and CNVnator uses RD variation. CNVnator was run with a bin size of 100 bp, as recommended by the authors of the software, and all other parameters were set to default values (Table 1, raw calls). For simplicity, we focus on duplications and do not report deletions in the resequenced individuals relative to the reference.

The three methods we used to generate our Discovery Sets (PE, RD and SRs) required mapping to a reference genome. Duplication of loci in the reference genome has been shown to influence the discovery of structural variants and the alignment strategy used is important in detecting duplications in repeated regions (Teo *et al.*, 2012). There were several different alignment strategies we could have chosen to deal with reads mapping to more than one location. It was possible to (1) discard these reads, (2) report all possible positions to which the reads map and (3) choose a position at random out of all equally good matching positions.

Limiting the analysis to uniquely mapped regions of the genome (strategy 1) would be likely to miss duplications, especially considering the high heterozygosity of these samples. Using algorithms that consider all possible mapping locations (strategy 2) has not been tested in samples where the mean RD is lower than 20 × (Teo *et al.*, 2012). All the samples we used to generate our Discovery Sets were sequence to an average of 15 × and hence we chose not to use this strategy. Placing a read at random when all the possible positions are an equally good match (strategy 3) has been shown to dilute the signal of duplications (Teo *et al.*, 2012). However, because this strategy has been used extensively in previous work and is a conservative strategy, we chose this over the other approaches (Zichner *et al.*, 2013).

### Filtering and merging duplication predictions: the discovery sets

**Figure 1:**
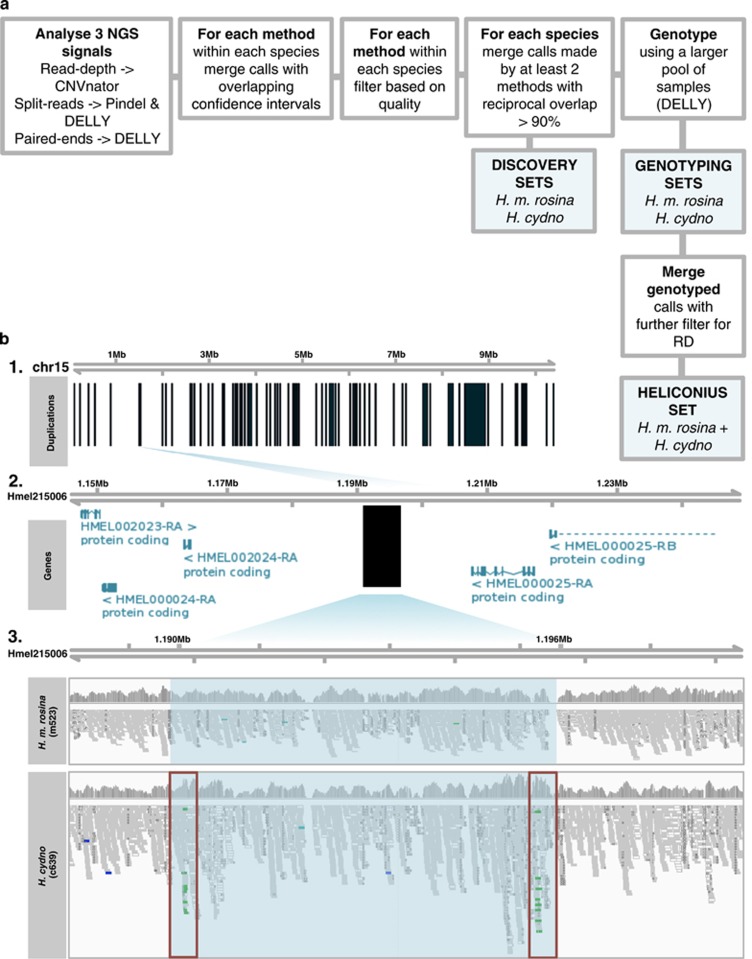
Duplication mapping and genotyping. (**a**) Integrated pipeline for duplication discovery (Discovery Sets) and genotyping (Genotyping Sets). Heliconius Set is the merged and filtered Genotyping sets from *H. cydno* and *H. melpomene*. (**b**) Example of a polymorphic duplication in *H. cydno* with respect to the *H. m. melpomene* reference genome (Davey *et al.*, 2016). (**b**1) Schematic representation of merged and genotyped Heliconius set duplication (vertical black rectangles) in Heliconius set for chromosome 15 (Table 1, Heliconius set). (**b**2) Zoom-in scaffold Hmel215006 to focus on a putative duplication from the merged genotyped set mapping 5′ end of the gene *cortex* (Nadeau *et al.*, 2016) (Table 3, Hmel215006:1190144-1196212). HMEL000025-RA and HMEL000025-RB are transcripts of *cortex* that map to Hmel215006:1205164-1324501. Genes flanking the duplication annotated as in Hmel2 (Davey *et al.*, 2016). (**b**3) Zooming-in further and looking at IGV RD and Illumina tracks for one *H. melpomene* and one *H. cydno* sample. Shaded light-blue region delineates the region that was identified as being duplicated. Red rectangles correspond to the breakpoint location of the region. Tracks are coloured green when a tandem duplication with respect to the reference genome is predicted by the read-pair orientation (PE) information.

To generate a list of non-redundant duplications for each species we combined the predictions generated by the three methods using custom scripts (available from Dryad) (Figure 1a). We calculated confidence intervals around each putative breakpoint according to the resolution defined for each method (DELLY: 50 bp outwards, 100 bp inwards; CNVnator: 1 kb outwards, 400 bp inwards; Pindel: +/−10 bp) (Zichner *et al.*, 2013) (Table 1, merged by tool; Figure 1a). We generated six duplication discovery call sets (one for each combination of three methods and two species) by combining all calls with overlapping confidence intervals at both start and end coordinates into a single event. Predictions made by DELLY had to have at least three read-pairs with a mapping quality higher than 20 supporting the call for each individual sample. We removed 311 duplication calls that were predicted by DELLY in all of the *H. melpomene* samples, and were therefore likely to represent either genome assembly errors or genuine deletions in the reference genome. Finally, we combined the three putative call sets within each species using the intansv module (v1.9.2) in R (v3.2.1; https://cran.r-project.org; Yao, 2015). We kept calls that had a reciprocal coordinate overlap of 90% or higher and were predicted by at least two methods. Previous studies had used an overlap of 80% (Zichner *et al.*, 2013). However, because the size and total count of the putative variants did not differ dramatically between cut offs of 80 and 90% in our data set (Supplementary Figures S1–S4), we chose to use 90% as a more conservative overlap parameter. This generated two species-specific duplication discovery call sets, one for *H. cydno* and one for *H. melpomene* (Table 1, Discovery Set; Figure 1a, Discovery Sets).

### Duplication genotype calling: the genotyping sets

To infer copy-number genotypes and evaluate the occurrence of each duplication in both Discovery Sets for all samples (20 *H. melpomene* and 14 *H. cydno*), we used the DELLY genotyper module with –t DUP option and default parameters (v0.7.2) (Rausch *et al.*, 2012). All duplications were treated as dominant loci and genotypes were scored as presence or absence in each sample. Using svprops, a program that computes various SV statistics from an input vcf file (https://github.com/tobiasrausch/svprops), we calculated median read support of each variant. We filtered out duplications with more than 500 reads mapping in an effort to discard repeats found at high copy number throughout the genome. We also filtered out events not genotyped in any of the samples, leaving high-quality Genotyping Sets of 497 putative duplications in *H. cydno* and 462 in *H. melpomene* (Figure 1a, Genotyping Sets).

### Merging the *H. melpomene* and *H. cydno* genotyping sets: the Heliconius set

There were 186 identified putative duplications in the Genotyping Set of *H. melpomene* and *H. cydno* with an overlap >90% and these were merged further using the intansv module (v1.9.2) in R (v3.2.1) (Yao, 2015). After merging both Genotyping Sets according to this criterion we produced the Heliconius Set (Figure 1). Each duplication event was treated as a dominant binary marker (0 for absence and 1 for presence). A duplication was considered to be absent (0) when individual *i* has the same number of copies of sequence *j* as the Hmel2 reference genome, whatever the number of *j* copies in the reference genome. Conversely, a duplication was considered to be present (1) when *i* has more copies of *j* than the Hmel2 reference genome. We called genotypes as presence/absence in this way, rather than calling heterozygotes (Rausch *et al.*, 2012).

### Inferring the quality of the putative calls by PacBio alignment and analysis of chromosome 2

**Figure 2:**
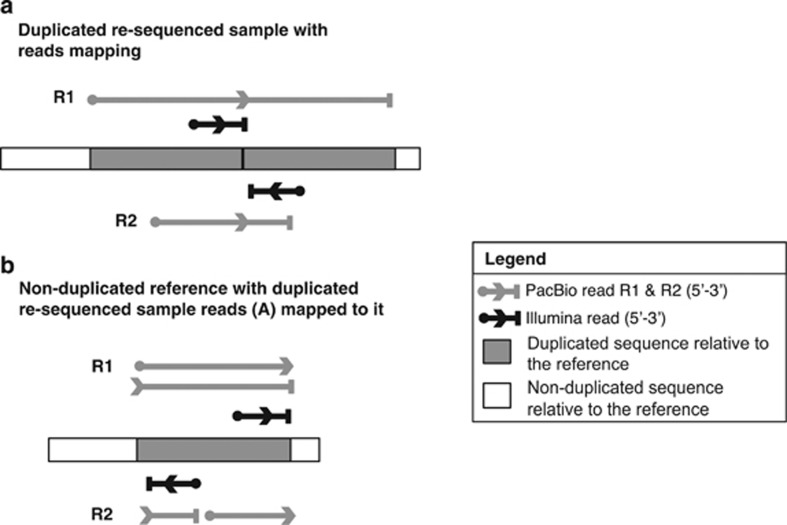
Validating short-read calls on chromosome 2 using PacBio single-molecule sequencing. Example of a breakpoint structure associated with a tandem duplication sequenced by Illumina chemistry (short reads, black) and PacBio chemistry (long reads, grey). A circle denotes the start of a read, the arrow its orientation, and the end is represented by a vertical bar. PacBio read R1 spans the entire duplicated sequence but PacBio read R2 does not. (**a**) Duplicated resequenced sample with Illumina and PacBio reads (R1 and R2) mapping. (**b**) Non-duplicated reference with duplicated resequenced sample reads from A mapped to it—tandem duplicated sequence aligned to a non-duplicated reference. Illumina reads from an individual with a tandem duplication map in divergent orientations when aligned to a reference without duplicated sequence. When PacBio read R1 is aligned to a non-duplicated reference, there are two alignments to the region that is flanked by the Illumina divergently oriented reads. The PacBio read R2 aligns discontinuously to the reference genome. The 3′ end of the R2 fragment of the breakpoint aligns to the reference upstream of the 5′ end of the R2 fragment.

We evaluated the accuracy of our duplication calling methods on a separate set of individuals for which appropriate long-read sequence data were available. These were one *H. melpomene* and one *H. cydno* family, for which the parents and one offspring from each family had been sequenced on an Illumina HiSeq 2000 (125 bp paired end, ENA accession ERP009507; see Malinsky *et al.*, 2016 for details). Our full duplication detection pipeline was run on these six individuals for chromosome 2. In addition, pools of 12 female and 12 male larvae from the same two families were sequenced on a Pacific Biosciences (PacBio, Menlo Park, CA, USA) RS II machine (P6/C4 chemistry, ENA submission in progress; read depths: *H. melpomene* females, 54x; *H. melpomene* males, 37x; *H. cydno* females, 49x; *H. cydno* males, 14x). Pacific Biosciences sequences were aligned to the *H. melpomene* reference genome version 2.0 (Davey *et al.*, 2016) with bwa mem (Li, 2013), using the PacBio option (-x). We then followed Layer *et al.* (2014) to validate our putative duplications, using sambamba (v0.6.1, Tarasov *et al.*, 2015) to select and filter the SRs from each PacBio bam file and converting these to the bedpe format (v2.25.0) (Quinlan and Hall, 2010) using the LUMPY (https://github.com/arq5x/lumpy-sv) custom script splitReadSamToBedpe. To convert the SRs to breakpoint calls we ran the custom script splitterToBreakpoint on each bedpe file with slope 1000 and default options for all other parameters (Layer *et al.*, 2014). The bedpe files with breakpoint information were merged for each species using bedtools intersectBed (v2.25.0) (Quinlan and Hall, 2010). We selected those reads that overlapped the start and end of the putative breakpoints called using Illumina short-read data. A putative duplication was considered validated when there were split long-read alignments within the predicted breakpoint interval such that (1) two segments of a single PacBio subread aligned to overlapping sections of the reference (Figure 2, PacBio read R1); or (2) if a single read aligned in split formation with the downstream end of the read aligning to a region that is upstream in the reference (Figure 2, PacBio read R2) (Layer *et al.*, 2014; Rogers *et al.*, 2014).

### Using the putative genotyping duplication call set to show population structure and differentiation

**Figure 3:**
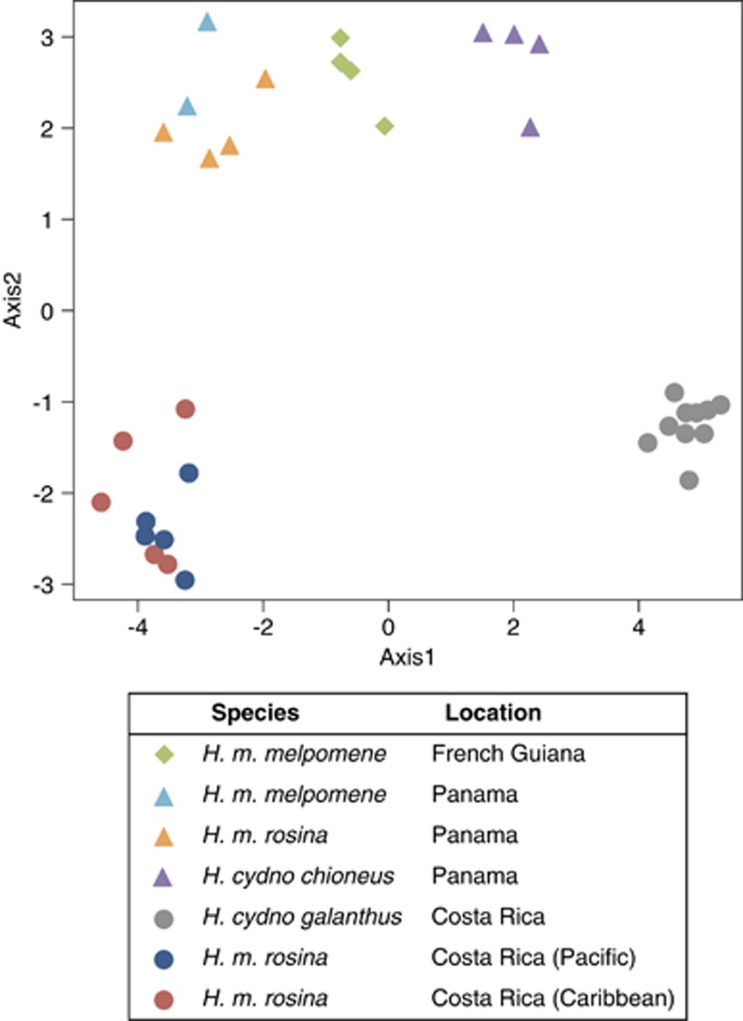
Principal component analysis of the duplicated variants in the Heliconius set. Samples cluster by species and location based on their duplication genotype. Of the total variance, 17.57% was explained by the first two principal components (PC1 12.97% and PC2 4.6%).

Putative duplications from the Heliconius Set were analysed as dominant loci by principal component analysis in using the R package adegenet (v1.3-1) (Figure 3; Armengol *et al.*, 2009; Jombart and Ahmed, 2011).

### Overlap between structural variants and genomic features

We investigated the overlap between the genotyped duplications and four different genomic features (genes, coding sequences (CDSs), introns and untranslated regions (UTRs)) using the R package ‘intervals' in both Genotyping sets (Figure 1a and Table 1 Genotyping set). A single duplication could fall into several subcategories. All duplications that overlapped with coding sequence were counted as CDS duplications. A duplication was considered to be intronic if it overlapped with an intron but not CDS. UTRs were considered in the same way as introns if it does not overlap with CDS. Overlap with any of these features was considered a gene-overlapping duplication. As a small number of the genotyped duplications were overlapping, these were merged for this analysis, so that only non-overlapping duplication intervals were considered. To investigate whether the observed number of duplications overlapping each class of genomic features was significantly larger or smaller than expected by chance, we simulated 10 000 randomized distributions of duplications across the genome. In each simulation, the defined set of duplication intervals (with overlapping intervals merged for simplicity) was randomly permuted into non-overlapping locations across the genome, and the number overlapping with each class of genomic feature was recorded. We used the 2.5 and 97.5% quantiles of the simulated distribution as critical values to assess whether the observed overlaps differed significantly from that expected under a random distribution of duplications.

### Detection of enriched biological functions within the Heliconius Set

We used InterProScan (v5.18.57.0; https://www.ebi.ac.uk/interpro/) (options –t n –goterms) to compare the Heliconius Set against the InterPro database. The InterPro database integrates predictive information from a number of sources (Mitchell *et al.*, 2015). We analysed PANTHER (http://www.pantherdb.org) database IDs that can be used to infer the function of uncharacterized genes based on their evolutionary relationships to genes with known functions (Mi *et al.*, 2016). We ran the PANTHER overrepresentation test on the Heliconius Set using the *D. melanogaster* genome as the reference list. We performed this analysis on the PANTHER GO-Slim Biological Process. We used the Bonferroni correction for multiple testing and report those categories overrepresented with *P*<0.05 (Supplementary Table S2 and Supplementary Figure S13). Five hundred and twenty nine overrepresented occurrences did not have a biological process associated with them but we have reported their predicted family name (Supplementary Table S3).

### Identifying outlier loci from the Heliconius Set

Duplications present in the Heliconius Set were tested for signals of divergent selection by identifying *F*_ST_ outliers using BayeScan (v2.1; Foll and Gaggiotti, 2008) with default parameters except that prior odds were set to 1 (Cheang *et al.*, 2013). *F*_ST_ was estimated for the Heliconius Set between (1) *H. cydno* Costa (Rica and Panama); and (2) *H. melpomene* (Costa Rica, Panama and French Guiana). Each duplication event was treated as a dominant binary marker (0 for absence and 1 for presence). We corrected for false positives (false discovery rate of *P*<0.05). Duplications with log posterior odds >1 have strong support for selection.

We also applied a related method that identifies loci subject to selection taking into account associated population/species-specific covariates, using BayPass v2.1 (http://www1.montpellier.inra.fr/CBGP/software/baypass/), for the putative duplications in the Heliconius Set (Gautier, 2015). The duplication events were considered as dominant binary markers. We used country coordinates and species as population-specific covariates. The covariates were defined as follows: Costa Rica: 9.7489, 83.7534; Panama: 8.5380, 80.7821; French Guiana: 3.9339, 53.1258; *H.* cydno: 1 and H*. melpomene*: 2. Under the Standard Covariate Model we estimated for each duplication event the Bayes Factor, the empirical Bayesian *P*-value and its underlying regression coefficient using an Importance Sampling algorithm. We simulated the data under the Inference Model to calibrate the neutral distribution of XtX. XtX was used to identify loci subjected to adaptive divergence. After calibrating XtX we ran the Markov chain Monte Carlo algorithm using posterior estimates available from the previous analysis and we corrected for location using just one covariable at a time, as suggested by Gautier (2015). Finally, we selected the duplication events that had observed XtX estimates above the 98% threshold of the simulated data (XtX >7.9). We cross-referenced the regions selected from BayeScan and BayPass analyses to look for overlaps between the two methods.

## Results

### Duplication maps for *H. cydno* and *H. melpomene*

**Figure 4:**
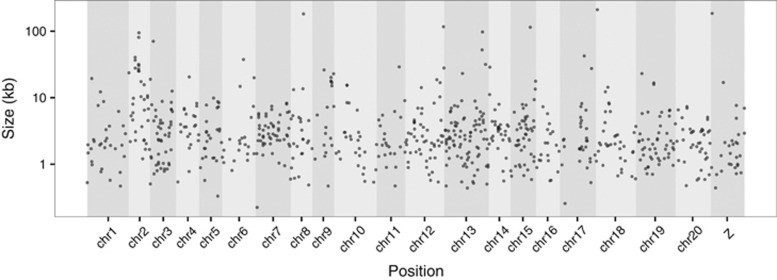
Distribution of the *Heliconius* duplication set mapped to the Hmel2 reference genome. *H. cydno* and *H. melpomene* genotyping sets were filtered and exclude duplications with a median read count of >500 reads per sample or not genotyped in any of the samples. The two high-quality genotyping sets were merged to produce the Heliconius duplication set (Heliconius Set, Figure 1a and Table 1). Each putative duplication on the Heliconius set is represented by a point according to position in the genome (x axis) and size (kb).

We identified a Discovery duplication set of 1920 putative *H. cydno* duplications and 1591 putative *H. melpomene* duplications (Table 1, Discovery set: merged by species) based on whole-genome resequencing data from 10 wild *H. cydno* samples and 10 wild *H. melpomene* samples (Kronforst *et al.*, 2013; Supplementary Table S1). We genotyped the discovery sets in a further 10 *H. melpomene* and 4 *H. cydno* samples (Martin *et al.*, 2013). After removing duplications with low-quality genotypes and high RD and duplications where all samples differed from the *H. melpomene* reference genome, we retained 497 putative *H. cydno* duplications and 463 *H. melpomene* duplications (Table 1, Genotyping set; Figure 4 and Supplementary Figures S5 and S6). We then merged redundant duplications in the *H. cydno* and *H. melpomene* Genotyping Sets, where two variants overlapped in over 90% of their total length, to produce the Heliconius Set containing 744 duplications ranging in size from 228 bp to 207 510 bp (median 5693 bp) (Table 1, Heliconius set; Supplementary Figures S7–S9).

### Validation rate as estimated by analysis of PacBio single-molecule long reads

We validated our pipeline using Illumina and PacBio sequencing data for a single chromosome from two families of *H. melpomene* and *H. cydno*. We first ran our pipeline on the Illumina data for chromosome 2 and then validated the calls using the PacBio data. Using the Illumina sequenced trio, we identified 97 duplications on chromosome 2 in *H. melpomene* and 137 in *H. cydno* after filtering. We validated 96.9% of the *H. melpomene* and 95.6% of the *H. cydno* calls using single-molecule PacBio SRs for each species separately. We also ran the Heliconius Set of duplications using the same PacBio data, combining the data from *H. cydno* and *H. melpomene*. This confirmed 65.5% of putative duplications. The lower validation rate on the Heliconius Set duplications is because of the fact that these are different individuals and populations compared with our PacBio data. In the Heliconius set a third to a quarter of all duplications identified only occurred in a single individual and hence were unlikely to be present in the PacBio data (Supplementary Figure S8). Nonetheless, the high validation observed in our reference trios suggests that our pipeline is correctly identifying duplications from Illumina data.

### Effect of genome structure on duplication distribution

Most duplications occurred in a small number of samples and there were only a few duplications at high frequency among all the samples (Supplementary Figure S8). For example, in the *H. cydno* genotyping set, 26.8% of the duplications are singletons and, in the *H. melpomene* 32.5%. The number of duplications per chromosome in the Heliconius Set is not equally distributed along the different chromosomes (Supplementary Figure S9A) and is weakly correlated with chromosome size (*r*^2^=0.344; Supplementary Figure S9B). There was also variation between individual chromosomes in the number of duplications per Mb (F(20,723)=14.2, *P*<0.001). Chromosome 18 tended to have fewer duplications, whereas chromosome 17 showed an excess of duplications per Mb compared with other chromosomes (*post hoc* Tukey's HSD (honest significant difference) test with correction for multiple testing). We did not observe any excess or depletion of duplication events towards the centres of chromosomes in the Heliconius Set (Supplementary Figure S10).

### Principal component analysis of the genotyped *H. cydno* and *H. melpomene* sets

We tested for population structure in the Heliconius Set of duplications genotyped as co-dominant markers using principal component analysis. In total, 17.57% of the total variance was explained by the first two principal components (PCs; PC1 12.97% and PC2 4.6%). Along PC1 the samples separated by species and geography (Figure 3), with all populations distinct except *H. m. melpomene* and *H. m. rosina* samples from Panama that are known to be genetically very similar (Martin *et al.*, 2013). However, PC2 separates the Costa Rica samples from those from Panama and French Guiana. It seems most likely that this is a methodological artefact because samples from different countries came from different sequencing runs (Supplementary Table S1). In addition, our call set was generated from the Costa Rica data set, and subsequently genotyped on both sample sets. Within Costa Rica, PCA analyses separate populations by geography and species as expected (Supplementary Figure S11).

### Overlap between duplication and genes

We found that the genotyped duplications in *H. melpomene* overlapped with genes and CDSs significantly less often than expected by chance, whereas the rate of overlap with UTRs and introns did not differ from the null expectation under a random distribution (Table 2 and Supplementary Figure S12). This is consistent with the idea that duplications involving functional regions have a greater probability of being deleterious, and are therefore more likely to be removed by selection. In contrast to *H. melpomene*, in *H. cydno*, there was no significant deviation from the null expectation in the rate of overlap between genotyped duplications and genes, CDSs, UTRs or introns.

### Enrichment of biological functions in the Heliconius Set

The duplications we have identified are not equally distributed across the genome (Figure 4 and Supplementary Figure S9). The heterogeneity observed across the landscape is likely to be a reflection of biases in the rates at which duplications arise in certain regions or a bias in the preservation of duplications in specific functional classes because of the action of natural selection. It has been shown that multigene families, specifically those involved in environmental responses, are particularly prone to being duplicated/retained (Duvaux *et al.*, 2015). We detected 19 gustatory receptors that had been previously identified as putatively duplicated by CNVnator analysis (Briscoe *et al.*, 2013). Moreover, we tested whether any biological functions were overrepresented in the Heliconius set of duplications using PANTHER (Supplementary Figure S13). Within the *Heliconius* set there were 1710 different family classes of which 1181 were associated with predicted biological processes. Of these processes, 26 different biological function categories were identified as overrepresented in the *Heliconius* set based on the *D. melanogaster* reference list (*P*<0.005) (Supplementary Figure S13 and Supplementary Table S2). These were involved in transketolase, phosphatase, endodeoxyribonuclease, metallopeptidase, lipid transport, deacetylase, oxidoreductase and transferase activity. There was also a set of 529 family classes that are overrepresented in the Heliconius set but do not have a specific Gene Ontology (GO) term, biological or specific molecular function associated with them but include ejaculatory bulb-specific protein, male sterility protein, cuticle formation and transposable element related (Supplementary Figure S13, Unclassified; Supplementary Table S3). Structural constituents of the cytoskeleton, protein binding, DNA binding transcription factor and kinase activity were molecular function categories underrepresented in the *Heliconius* set. The biological function that was most overrepresented in the entire set was the GO category related to the pentose-phosphate shunt (primary metabolic process, fold enrichment 18.35, *P*=5.4e–07). Immune system processes were underrepresented in our set (fold enrichment <0.2, *P*=2.59e–04).

### Identification of outlier duplications in the Heliconius Set potentially under selection

To characterize patterns of divergence observed between *H. melpomene* and *H. cydno* we first calculated *F*_ST_ between the two species and identified candidate outlier regions using BayeScan for the Heliconius Set of duplications, treating putative duplications as co-dominant (presence/absence) markers. After correcting for false positives we found nine duplications that are candidates for selection (Supplementary Figure S14A and Supplementary Table S4). We also ran BayPass that conducts a similar test by accounting for sample location and species. This produced six putative duplicated regions above the simulated significance threshold (Supplementary Figure S14B and Supplementary Table S4), four of which were also identified by BayeScan (Table 3). We consider the four outlier events found by both tests to be strong candidates for directional selection. One region, on chromosome 15, is located in an intergenic region upstream of the gene *cortex* that is involved in the regulation of yellow and white wing pattern elements (Figure 1b) (Nadeau *et al.*, 2016). The other three regions overlap with genes, predicted to be a Kazal-type serine protease (chromosome 9), an odorant binding protein (chromosome 18) and a regulator of the cell cycle and nitrogen compound metabolic processes (chromosome 21) (Table 3). All four candidate selected duplications are absent in the *H. melpomene* samples and present in 13 or 14 of the 14 *H. cydno* samples.

## Discussion

Gene duplication is an important source of genetic fuel for evolutionary diversification, and can also contribute to speciation. Here we have used short-read genome sequence data to identify signatures of CNV in natural populations. We have used single-molecule sequencing to validate our pipeline, with a validation rate of ~96% within families. We have successfully identified 744 loci and genotyped them (presence/absence) in 34 wild individuals sampled from the two species *H. melpomene* and *H. cydno*.

Despite the ubiquitous nature of duplications, different chromosomes might be expected to contribute differently to the overall duplication landscape. Large chromosomes tend to have the highest absolute duplication counts but chromosome size is not the sole predictor of duplication distributions. Sex chromosomes, which have more repetitive content, smaller population sizes and lower levels of background selection than autosomes, have been shown to have a higher duplication load per base pair than autosomes in *D. simulans* and in *D. melanogaster* (Charlesworth, 2012; Mackay *et al.*, 2012; Zichner *et al.*, 2013; Rogers *et al.*, 2014, 2015). However, the X chromosome of *Drosophila yakuba* does not contain an excess of duplications compared with the autosomes and no signals of adaptation through duplication have been identified. Similarly, the *Heliconius* duplication set does not harbour an excess of duplications on the Z chromosome compared with the autosomes. It is possible that duplications are more difficult to detect on the Z chromosome that has higher divergence than the rest of the genome (Martin *et al.*, 2013) and higher proportion of repetitive content (Conrad and Hurles, 2007). Further work will be needed to compare the landscape of duplications across sex chromosomes.

Duplications are not homogenously distributed across the genome (Figure 2 and Supplementary Figures S5 and S6). There was no bias towards telomeric regions as has been documented for humans (Zhang *et al.*, 2005). *Heliconius*, like *C. elegans*, have holocentric chromosomes and, to our knowledge the enrichment of structural variations in telomeric regions (and/or pericentrimeric regions) has yet to be documented for organisms with this chromosomal organization (Farslow *et al.*, 2015). The number of singletons identified in our data set (a quarter to a third of all duplications) is on the same order of magnitude as that seen previously. For example, Duvaux *et al.* (2015) reported 31% singletons in pea-aphid clones.

A large proportion of structural variants arising in genomes are slightly or moderately deleterious and therefore experience purifying selection (Emerson *et al.*, 2008; Zichner *et al.*, 2013). In *D. melanogaster*, fewer duplications were found in coding sequence as compared with random expectation (Zichner *et al.*, 2013). Consistent with this, we found that in the *H. melpomene* Genotyping Set duplications are biased away from coding regions, although they are not biased away from or towards intronic or UTR regions. However, we did not find a similar bias in *H. cydno*, and saw no significant depletion of the number of duplications in *H. cydno* as compared with *H. melpomene*. This goes against expectations, given that the effective population size of H. *cydno* has been inferred to be around four times greater than that of *H. melpomene* (Kronforst *et al.*, 2013), consistent with the significantly higher genome-wide heterozygosity in *H. cydno* (Martin *et al.*, 2013). Therefore, we might expect selection to operate more effectively and duplications to be more efficiently removed from *H. cydno*, but this does not appear to be the case. We do not have any good explanation for this.

Although most structural variants may be deleterious, there is particular interest in those few that have positive effects. There are now many examples in which gene duplicates provide the genetic fuel for adaptation, and have been shown to be under positive selection (Beisswanger and Stephan, 2008; Arroyo *et al.*, 2012; Blount *et al.*, 2012). Here, we are specifically interested in speciation. Gene duplicates have been implicated in reproductive isolation for both animals and plants. For example, the Odysseus gene that causes hybrid sterility between *D. mauritiana* and *D. simulans* is a duplicate of the *unc-4* gene (Ting *et al.*, 2004). In *A. thaliana*, paralogues of an essential duplicate gene that evolved divergently interact epistatically in some interspecific crosses and control a recessive embryo lethality (Bikard *et al.*, 2009). In the context of *Heliconius*, we are specifically interested in speciation and divergent selection between the closely related species, *H. melpomene* and *H. cydno*. Using BayeScan and BayPass we identified a relatively small number of duplications that are putatively divergently selected between these species.

Many functionally important regions in different genomes have been documented to evolve through gene duplication followed by neo or subfunctionalization. Genes responsible for environmental response are known to be overrepresented as duplicated sequences in a range of organisms from humans to fruit flies and butterflies (Johnson *et al.*, 2001; Tuzun *et al.*, 2005; Hahn *et al.*, 2007; Briscoe *et al.*, 2013) and in line with previous studies we have detected an enrichment of genes involved in sensory perception (Briscoe *et al.*, 2013; Rogers *et al.*, 2014; Duvaux *et al.*, 2015; Paudel *et al.*, 2015). For example, we detected gustatory receptors that had already been identified in *Heliconius* (Briscoe *et al.*, 2013) but we also detected others such as olfactory receptors and olfactomedin-related proteins (Supplementary Table S3). Specifically, in our outlier analysis there is an odorant binding protein that is divergent in copy number between *H. cydno* and *H. melpomene* (OBP41, Table 3). Several hypotheses have been put forward to explain the trend of increased CNV among genes involved in environmental response. On one hand, these CNVs might be maintained by positive selection as outlier analysis-based methods have shown an enrichment for these GO classes (Duvaux *et al.*, 2015; Paudel *et al.*, 2015; Rogers *et al.*, 2015). On the other hand, these differences could occur simply because certain sequence motifs like non-B DNA forming sequence are more common in gene-rich regions and, at the same time, they increase the rate of CNV formation (Sjödin and Jakobsson, 2012). Gene categories overrepresented in CNV are also enriched within segmental duplications, and segmental duplications are very structurally dynamic (Conrad and Hurles, 2007). Moreover, families with multiple paralogues are more prone to further copy number variation (Hastings *et al.*, 2009).

Not all the putative duplications we found as outliers were involved in environmental response. Another candidate locus under divergent selection was found near the *cortex* gene that controls the yellow hindwing bar and white/yellow forewing patterns that differ between *H. m. rosina* and *H. cydno* (Nadeau *et al.*, 2016). Moreover, we have also found an enrichment of male reproductive proteins in the Heliconius Set (Supplementary Table S3). These proteins evolve rapidly and are commonly duplicated in, for example, *D. yakuba* (Rogers *et al.*, 2014). It was somewhat surprising, however, that we did not observe an enrichment for immunity-related genes.

Interestingly, the four putative duplicated regions we have identified as excessively differentiated in *H. cydno* and *H. melpomene* were all nearly fixed in *H. cydno* but not in *H. melpomene. H. melpomene* and *H. cydno* differ in many aspects of their ecology and behaviour. Shifts in host plant have played a central role in their diversification. The evolution of host-use strategies reflects a tradeoff between selection pressures (Merrill *et al.*, 2013). For example, gene duplications that persist in an evolving lineage have often been found to be beneficial because of a protein dosage effect in response to environmental conditions. Host-plant systems may be subject to rapid coevolution and duplicated loci in *H. cydno* could be related to the fact that *H. cydno* is a host plant generalist and *H. melpomene* is a specialist (Merrill *et al.*, 2013).

The duplications we have identified as being under selection between *H. cydno* and *H. melpomene* may play a role in species divergence. We have shown that, despite being ubiquitous, the landscape of duplications in *Heliconius* is heterogeneous and likely to be under both positive and negative selection. The putative duplications we found merit further investigation for their potential role in host plant and mate recognition differences between the species.

## Data archiving

All short-read sequence data are publicly available (Kronforst *et al.*, 2013; Martin *et al.*, 2013; Malinsky *et al.*, 2016). Long-read Pacific Biosciences data are available at European Nucleotide Archive accession PRJEB6424. Custom scripts, Genotyping Sets and Heliconius Set are available from Dryad (10.5061/dryad.8jv30).

## Figures and Tables

**Table 1:**
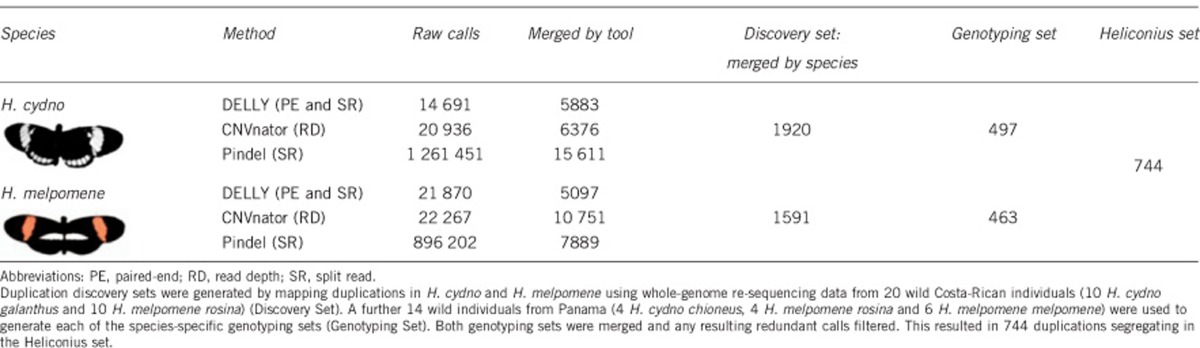
Duplication discovery and genotyping in *Heliconius cydno* and *Heliconius melpomene*

**Table 2:** Functional impact of the Heliconius set

*Species*	*Complete gene*	*%*	*< Sim 2.5%*	*Gene*	*%*	*<Sim 2.5%*	*CDS*	*%*	*<Sim 2.5%*	*Intron*	*%*	*<Sim 2.5%*	*UTR*	*%*	*<Sim 2.5%*
*Heliconius melpomene*	23	5.2	No	157	35.3	Yes	92	20.7	Yes	45	10.1	No	27	6.1	No
*Heliconius cydno*	41	8.9	No	210	45.8	No	154	33.6	No	42	9.2	No	20	4.4	No

Abbreviations: CDS, coding sequence; UTR, untranslated region.

Observed absolute counts and proportion of duplications overlapping complete genes, genes, CDS, introns and UTRs. <Sim 2.5% column indicates whether the observed proportion of overlap with each category falls within the 2.5% confidence interval of the simulated data overlap after 10 000 iterations. If <sim 2.5% is ‘No', then duplication counts are not within the 2.5% confidence interval and the overlaps observed do not significantly differ from random expectations. If ‘Yes', then counts are within the 2.5% confidence interval and the overlap observed is significantly less than expected under a random distribution. A single duplication can fall into several subcategories.

**Table 3:** Putative duplicated loci under selection between *Heliconius cydno* and *Heliconius melpomene*

*Chr*	*Scaffold*	*Start*	*End*	*Size*	*BayeScan log10(PO)*	*BayPass mean XtX*	*Freq in H. melpomene*	*Freq in H. cydno*	*PANTHER GO-Slim Biological process*	*Hmel2 annotation*
9	Hmel209007	4 344 840	4 364 959	20 119	1.7222	7.95239143	0	0.93	Kazal-type serine protease inhibitor	HMEL009267
15	Hmel215006	1 190 144	1 196 212	6068	1.8414	8.78515118	0	1	NA	upstream of *cortex*
18	Hmel218003	221 730	42 9239	207 509	1.894	8.75630075	0	1	Protein targeting Intracellular protein transport Transport Localization Biological regulation Asymmetric protein localization	OBP41 HMEL013558 HMEL013559 HMEL003174 HMEL003175 HMEL003862 HMEL003863
21	Hmel221012	779 541	796 444	16 903	1.72	8.35788884	0	0.93	Regulation of the cell cycle Regulation of biological process Porphyrin-containing compound Metabolic process Nitrogen compound metabolic process Regulation of translation Primary metabolic process mRNA transcription Nucleobase-containing compound metabolic process Cell differentiation, developmental process Regulation of transcription from RNA pol II promoter	HMEL016617 HMEL016621 HMEL016620

Abbreviation: NA, not available.

The four duplications in the Heliconius set identified as outliers by BayeScan and BayPass analysis. Chromosome position, scaffold name, start, end and size of each putative duplication are indicated. log10 (Posterior Probabilities) from the BayeScan analysis is indicated per duplication between the *H. melpomene* and *H.* cydno. All these loci had positive values of α that suggests diversifying selection. BayPass XtX mean for each loci is also indicated for each species after correcting for location. Allele frequencies calculated as co-dominant markers are shown for each species at the loci (genotyped by Delly2). PANTHER GO-Slim biological processes and Hmel2 annotations retrieved from Hmel2.gff (Davey *et al.*, 2016).
