# Supplementary material for: The comparative landscape of duplications in Heliconius melpomene and Heliconius cydno
Source: Heredity (Edinb). 2016 Dec 7;118(1):78–87. doi: 10.1038/hdy.2016.107 (PMC5176112; doi:10.1038/hdy.2016.107)
Supplement: Supplementary Figures [file hdy2016107x1.docx]

**Supplementary Figures with legends**

**Figure S1.**

**Figure S1. Size Distribution of duplication calls depending on merging overlapping criteria for *H. melpomene***

Depending on the chosen overlap percentage median sizes and distributions of duplications in the *H. melpomene* Discovery Set varies.

**Figure S2.**

**Figure S2. Size Distribution of duplication calls depending on merging overlapping criteria for *H. cydno***

Depending on the chosen overlap percentage median sizes and distributions of duplications in the *H. cydno* Discovery Set varies.

**Figure S3.**

**Figure S3. Density distribution of duplication calls depending on merging overlapping criteria for *H. melpomene***

Depending on the chosen overlap percentage median sizes and distributions of duplications in the *H. melpomene* Discovery Set varies.

**Figure S4.**

**Figure S4. Density distribution of duplication calls depending on merging overlapping criteria for *H. cydno***

Depending on the chosen overlap percentage median sizes and distributions of duplications in the *H. cydno* Discovery Set varies.

**Figures S5.**

**Figure S5. Genotyping *H. cydno* set**

Genotyped duplication set in *H. cydno* with 497 duplications

**Figures S6.**

**Figure S6. Genotyping *H. melpomene* set**

Genotyped duplication set in *H. melpomene* with 463 duplications

**Figure S7.**

**Figure S7. Size distribution of the Genotyping *Heliconius cydno* and *H. melpomene* duplication sets**

Size distribution of the calls for the *H. cydno* and *H. melpomene* Genotyping Sets. *H. cydno* is represented in blue and *H. melpomene* in pink. Size in kb.

**Figure S8.**

**Figure S8. Variant allele counts for the *H. melpomene* and *H. cydno* Genotyping sets and variant allele frequency in the Heliconius set**

Variant allele frequency for the Heliconius Set for the 14 *H. cydno* and 20 *H. melpomene*. Duplication alleles also treated as co-dominant (presence/absence) markers.

**Figure S9.**

**Figure S9. Genome-wide distribution of duplications in the *Heliconius* set.** (A) Box-and-whisker plots displaying the number of inferred genotyped duplications per 1Mb-window for each chromosome. (B) Overall the number of duplications genotyped in the *Heliconius* set correlates with chromosome size. Each point represents one chromosome. Chromosomes that have a greater number of absolute duplications than the fitted line are also identified by their number above the point. Chromosome 21 (Z, sex-chromosome) has also been identified.

**Figure S10.**

**Figure S10. Distribution of duplications in the Heliconius set along chromosome position**

Number of duplications identified in the Heliconius set and their normalised distance from the chromosome centre. In the x axis -1 is the normalised chromosome position and 1 is the normalised chromosome end. 0 is the chromosome centre. Line fit to the correlation between normalised chromosome location and number of duplications.

**Figures S11.**

**Figure S11. Principal component analysis of the duplicated variants in the *Heliconius* set with Costa Rican samples.**

Samples cluster by species (PC1) and location (PC2) based on their duplication genotype. 23.47% of the total variance was explained by the first two principal components (PC1 18.856% and PC2 4.618%).

**Figure S12.**

**A.**

**B.**

**Figure S12. Observed and simulated proportions of duplications overlapping with coding regions**

The grey shading represents the distribution of simulated overlaps on 10,000 random replicates for the proportion of sites overlapping coding regions for duplications in *H. melpomene* (A) and *H. cydno* (B) (Table 2, Gene %). Vertical dotted lines indicate the mean and standard deviation for the overlap from 10 000 random simulations. The vertical dashed line indicates the observed percentage overlap of duplications with genic sequence for *H. melpomene* (A) and *H. cydno* (B) (Table 2, Gene %).

**Figure S13.**

| **ID** | **PANTHER GO-Slim Biological Process** |
| --- | --- |
| **1** | pentose-phosphate shunt (GO:0006098) |
| **2** | peroxisomal transport (GO:0043574) |
| **3** | vitamin biosynthetic process (GO:0009110) |
| **4** | cellular amino acid biosynthetic process (GO:0008652) |
| **5** | respiratory electron transport chain (GO:0022904) |
| **6** | DNA replication (GO:0006260) |
| **7** | vitamin transport (GO:0051180) |
| **8** | generation of precursor metabolites and energy (GO:0006091) |
| **9** | protein glycosylation (GO:0006486) |
| **10** | DNA metabolic process (GO:0006259) |
| **11** | proteolysis (GO:0006508) |
| **12** | steroid metabolic process (GO:0008202) |
| **13** | primary metabolic process (GO:0044238) |
| **14** | Unclassified (UNCLASSIFIED) |
| **15** | cellular process (GO:0009987) |
| **16** | biological regulation (GO:0065007) |
| **17** | RNA metabolic process (GO:0016070) |
| **18** | cell communication (GO:0007154) |
| 19 | transcription, DNA-dependent (GO:0006351) |
| 20 | regulation of biological process (GO:0050789) |
| 21 | developmental process (GO:0032502) |
| 22 | phosphate-containing compound metabolic process (GO:0006796) |
| 23 | response to stimulus (GO:0050896) |
| 24 | translation (GO:0006412) |
| 25 | response to stress (GO:0006950) |
| 26 | immune system process (GO:0002376) |
| 27 | protein phosphorylation (GO:0006468) |

**Figure S13. Enrichment of certain biological process classes in the Heliconius Set against the *D. melanogaster* PANTHER reference**

Fractional differences on the number of genes observed against the expected for each biological process category. Difference was calculate as the (number of genes observed for the category – number of gene expected for the category) / number gene expected for the category. Bars coloured by P value associated with each call, where P<0.05. Biological categories were given an ID from 1 to 27 from the one with the highest fold change to the one with the least. Negative values indicate a significant depletion genes associated with the biological process in question. Positive values an enrichment. PANTHER GO-slim Biological Process categories and the GO term associated with them shown below the fractional difference analysis.

**Figure S14.**

**A.**

**B.**

**Figure S14. Scan for selection on genomic regions putatively duplicated**

A) BayeScan outlier analysis on the Heliconius duplication set using 14 *H. cydno* and 20 *H. melpomene* samples genotyped as dominant markers. 9 out of the 744 identified as putative duplications are expected to be under divergent selection between *H. cydno* and *H. melpomene* (FDR<0.05, shown in orange). log(PO), posterior odds score. B) BayPass outlier analysis on the Heliconius Set as dominant markers. Analysis was performed using the following covariates Costa Rica: 9.7489, 83.7534; Panama: 8.5380, 80.7821; French Guiana: 3.9339, 53.1258; *H.* cydno: 1 and H*. melpomene*: 2. Horizontal line represents the 98% threshold of the simulated data (XtX>7.9). Points above the threshold correspond to those duplications regions identified by the outlier analysis after correcting for location. x axis plots each duplication in the Heliconius set (744 duplications in total). y axis represents the mean XtX for the region.
