# Supplementary material for: The comparative landscape of duplications in Heliconius melpomene and Heliconius cydno
Source: Heredity (Edinb). 2016 Dec 7;118(1):78–87. doi: 10.1038/hdy.2016.107 (PMC5176112; doi:10.1038/hdy.2016.107)
Supplement: Supplementary Table S1 [file hdy2016107x2.pdf]

ID	Submission	Accession number	Taxon	Sex	Country	Latitude	Longitude	Sequencing Center
c511	SRA106228	SRR1057584	*H. cydno galanthus*	Female	Costa Rica	10°16' N	84°11'W	BGI
c512	SRA106228	SRR1057585	*H. cydno galanthus*	Male	Costa Rica	9°40'N	83°2'W	BGI
c513	SRA106228	SRR1057586	*H. cydno galanthus*	Male	Costa Rica	10° 26'N	83° 59'W	BGI
c514	SRA106228	SRR1057587	*H. cydno galanthus*	Female	Costa Rica	9°43'N	83°3'W	BGI
c515	SRA106228	SRR1057588	*H. cydno galanthus*	Female	Costa Rica	10° 13'N	83° 41'W	BGI
c563	SRA106228	SRR1057589	*H. cydno galanthus*	Male	Costa Rica	10° 13'N	83° 47'W	BGI
c614	SRA106228	SRR1057590	*H. cydno galanthus*	Male	Costa Rica	9°43'N	83°3'W	BGI
c630	SRA106228	SRR1057591	*H. cydno galanthus*	Female	Costa Rica	10° 26'N	83° 59'W	BGI
c639	SRA106228	SRR1057592	*H. cydno galanthus*	Male	Costa Rica	10° 13'N	83° 41'W	BGI
c640	SRA106228	SRR1057593	*H. cydno galanthus*	Female	Costa Rica	9°43'N	83°3'W	BGI
m523	SRA106228	SRR1057594	*H. melpomene rosina*	Male	Costa Rica	9°43'N	83°3'W	BGI
m524	SRA106228	SRR1057595	*H. melpomene rosina*	Female	Costa Rica	9°51'0N	84°19'W	BGI
m525	SRA106228	SRR1057596	*H. melpomene rosina*	Male	Costa Rica	8°28'N	83°35'W	BGI
m589	SRA106228	SRR1057597	*H. melpomene rosina*	Male	Costa Rica	9°52'N	83°0'W	BGI
m675	SRA106228	SRR1057598	*H. melpomene rosina*	Male	Costa Rica	9°24'N	84°10'W	BGI
m676	SRA106228	SRR1057599	*H. melpomene rosina*	Male	Costa Rica	9°43'N	83°3'W	BGI
m682	SRA106228	SRR1057600	*H. melpomene rosina*	Male	Costa Rica	10°26'N	83°59'W	BGI
m683	SRA106228	SRR1057601	*H. melpomene rosina*	Male	Costa Rica	9°24'N	84°10'W	BGI
m687	SRA106228	SRR1057602	*H. melpomene rosina*	Female	Costa Rica	10°26'N	83°59'W	BGI
m689	SRA106228	SRR1057603	*H. melpomene rosina*	Female	Costa Rica	9°51'0N	84°19'W	BGI
247-1	ERA206886	ERR260277	*H. melpomene rosina*	Male	Panama	9°1206'N	79°6969'W	The GenePool
247-2	ERA206886	ERR260278	*H. melpomene rosina*	Male	Panama	9°1206'N	79°6969'W	The GenePool
247-3	ERA206886	ERR260279	*H. melpomene rosina*	Male	Panama	9°1206'N	79°6969'W	The GenePool
248-4	ERA206886	ERR260280	*H. melpomene rosina*	Male	Panama	9°1206'N	79°6969'W	The GenePool
248-5	ERA206886	ERR260281	*H. melpomene melpomene*	Male	French Guiana	4°9632'N	52°4200'W	The GenePool
248-6	ERA206886	ERR260282	*H. melpomene melpomene*	Male	French Guiana	4°9632'N	52°4200'W	The GenePool
248-7	ERA206886	ERR260283	*H. melpomene melpomene*	Male	French Guiana	4°9632'N	52°4200'W	The GenePool
249-8	ERA206886	ERR260284	*H. melpomene melpomene*	Male	French Guiana	4°9151'N	52°3755'W	The GenePool
249-9	ERA206886	ERR260285	*H. melpomene melpomene*	Female	Panama	8°6136'N	78°1398'W	The GenePool
249-10	ERA206886	ERR260286	*H. melpomene melpomene*	Male	Panama	8°2797'N	77°8098'W	The GenePool
252-19	ERA206886	ERR260295	*H. cydno chioneus*	Male	Panama	9°1714'N	79°7573'W	The GenePool
252-20	ERA206886	ERR260296	*H. cydno chioneus*	Male	Panama	9°1714'N	79°7573'W	The GenePool
252-21	ERA206886	ERR260297	*H. cydno chioneus*	Male	Panama	9°1714'N	79°7573'W	The GenePool
252-22	ERA206886	ERR260298	*H. cydno chioneus*	Male	Panama	9°1714'N	79°7573'W	The GenePool

Mean read-depth	Total raw reads	Mapped reads	Unmapped reads
14.57	56980695	54904440	2076255
14.67	57786331	55444921	2341410
14.83	58047481	56045188	2002293
14.73	57576892	55612600	1964292
14.70	57606552	55532956	2073596
13.83	54029777	52103112	1926665
14.81	57793383	55949422	1843961
13.83	54218576	52122166	2096410
14.84	57839669	55879865	1959804
14.82	57905245	55775583	2129662
14.94	58408944	55702153	2706791
15.03	57327760	55904796	1422964
14.58	58361427	54333551	4027876
14.92	56991140	55771647	1219493
15.07	57387640	56029868	1357772
13.63	53924476	50738856	3185620
14.96	57455142	55639521	1815621
14.19	54047170	52772924	1274246
15.06	57500817	56116208	1384609
14.71	57496048	54767875	2728173
26.9	80357189	77576309	2780880
26.7	80780396	77202364	3578032
26.5	79483045	76412158	3070887
36.7	109113325	106534913	2578412
24.1	89838758	71357196	18481562
23.1	83950002	68504773	15445229
35.0	107926916	104283723	3643193
35.8	110399158	106326491	4072667
62.0	190117929	184629062	5488867
15.6	47146484	44511911	2634573
35.8	111609395	105025362	6584033
35.3	108838440	103731516	5106924
39.2	119944299	115366463	4577836
46.0	146341596	135597591	10744005
