# Supplementary material for: The comparative landscape of duplications in Heliconius melpomene and Heliconius cydno
Source: Heredity (Edinb). 2016 Dec 7;118(1):78–87. doi: 10.1038/hdy.2016.107 (PMC5176112; doi:10.1038/hdy.2016.107)
Supplement: Supplementary Table S2 [file hdy2016107x3.pdf]

ID	PANTHER_GO-Slim_Biological_Process	D.melanogaster-REFLIST(13690)	HeliconiusSet(959)
1	pentose-phosphate_shunt(GO:0006098)	7	9
2	peroxisomal_transport(GO:0043574)	14	8
3	vitamin_biosynthetic_process(GO:0009110)	27	9
4	cellular_amino_acid_biosynthetic_process((	61	20
5	respiratory_electron_transport_chain(GO:0(	238	64
6	DNA_replication(GO:0006260)	86	23
7	vitamin_transport(GO:0051180)	50	13
8	generation_of_precursor_metabolites_and_	287	64
9	protein_glycosylation(GO:0006486)	98	21
10	DNA_metabolic_process(GO:0006259)	232	46
11	proteolysis(GO:0006508)	606	111
12	steroid_metabolic_process(GO:0008202)	159	26
13	primary_metabolic_process(GO:0044238)	4149	355
14	Unclassified(UNCLASSIFIED)	7319	529
15	cellular_process(GO:0009987)	2878	134
16	biological_regulation(GO:0065007)	1560	69
17	RNA_metabolic_process(GO:0016070)	1039	41
18	cell_communication(GO:0007154)	914	35
19	transcription,_DNA-dependent(GO:0006351	729	27
20	regulation_of_biological_process(GO:00507	1104	35
21	developmental_process(GO:0032502)	546	17
22	phosphate-containing_compound_metaboli	539	16
23	response_to_stimulus(GO:0050896)	739	19
24	translation(GO:0006412)	387	9
25	response_to_stress(GO:0006950)	285	4
26	immune_system_process(GO:0002376)	288	4
27	protein_phosphorylation(GO:0006468)	266	2

HeliconiusSet(expected)	(observed-expected)/expected	HeliconiusSet(over/under)	HeliconiusSet(foldEnrichment)	HeliconiusSet
0,49	17,36734694	+	18.35	5.40E-07
0,98	7,163265306	+	8.16	1.67E-03
1,89	3,761904762	+	4.76	2.96E-02
4,27	3,683840749	+	4.68	5.01E-06
16,67	2,839232154	+	3.84	5.72E-17
6,02	2,820598007	+	3.82	1.79E-05
3,5	2,714285714	+	3.71	1.41E-02
20,1	2,184079602	+	3.18	3.74E-13
6,87	2,056768559	+	3.06	1.90E-03
16,25	1,830769231	+	2.83	1.46E-07
42,45	1,614840989	+	2.61	2.33E-17
11,14	1,333931777	+	2.33	1.71E-02
290,64	0,221442334	+	1.22	9.94E-04
512,7	0,031792471	+	1.03	0.00E00
201,61	-0,335350429	-	.66	2.46E-06
109,28	-0,368594436	-	.63	1.75E-03
72,78	-0,436658423	-	.56	3.84E-03
64,03	-0,453381228	-	.55	6.37E-03
51,07	-0,471313883	-	.53	2.26E-02
77,34	-0,547452806	-	.45	3.87E-06
38,25	-0,555555556	-	.44	1.44E-02
37,76	-0,576271186	-	.42	8.12E-03
51,77	-0,63299208	-	.37	1.65E-05
27,11	-0,668019181	-	.33	8.62E-03
19,96	-0,799599198	-	.20	2.92E-03
20,17	-0,801685672	-	0.2	2.45E-03
18,63	-0,892646269	-	0.2	2.59E-04

t(P-value)
