# Supplementary material for: The comparative landscape of duplications in Heliconius melpomene and Heliconius cydno
Source: Heredity (Edinb). 2016 Dec 7;118(1):78–87. doi: 10.1038/hdy.2016.107 (PMC5176112; doi:10.1038/hdy.2016.107)
Supplement: Supplementary Table S3 [file hdy2016107x4.pdf]

Family ID	Mapped ID
PTHR10009	492
PTHR10353:SF36	342,89,387,673,558,826,608,361
PTHR10353	819,362,463,672,386,452,343,257,466,246,37[illegible]
PTHR10380	794
PTHR10492	473,113,830,832,848,508
PTHR10632:SF2	430,854
PTHR10632	431,855
PTHR10642	360
PTHR10695	132,638,716
PTHR10799	253,676,799,327,605,560
PTHR10989	833
PTHR11011:SF26	167,772,169,774,724,726,914,916
PTHR11011	168,773,775,725,170,727,915,917
PTHR11012	355,658,690
PTHR11012:SF5	689
PTHR11069	804,14
PTHR11117	487
PTHR11117:SF2	486
PTHR11257:SF2	561,485,661,148,9,171
PTHR11257	484,495,562,662,578,149,513,515,381,172,10
PTHR11257:SF5	512,579,514,382,494
PTHR11559	596,696,314,80,272,196,928,461
PTHR11610	310,245,37,679,736,936
PTHR11616	388,137,655,437,900,812
PTHR11920	165,793,164
PTHR11926	58,598
PTHR11950	642
PTHR12236	740,872,373
PTHR12236:SF15	873

PTHR12236:SF2		741
PTHR12236:SF6		372
PTHR12315		330,125
PTHR12315:SF0		124,329
PTHR12411	465,48,269,592	
PTHR12587:SF20	782,278,367,345,820,869,250,807	
PTHR12587	783,344,366,279,821,932,868,825,251,806	
PTHR12832	920,717,838	
PTHR12863		896,901
PTHR12863:SF1		902
PTHR13799	703,758,729	
PTHR13799:SF13	757,704,728	
PTHR13856		754,708
PTHR14445		432,489
PTHR14445:SF38		488
PTHR14517		940,315
PTHR14624		365,849
PTHR14677:SF2		334,337
PTHR14677		335,336
PTHR15863		851
PTHR15863:SF2		850
PTHR16089:SF29	189,630,888,509	
PTHR16089	188,510,889,629	
PTHR16441		422,39
PTHR16441:SF0		421
PTHR16453		737
PTHR18966		143
PTHR18966:SF188		142
PTHR19446:SF371		660
PTHR19446:SF364	444,427,174,581	

PTHR19446:SF376	503
PTHR19446:SF244	451,275,441,632,720,280,185,87
PTHR19446:SF355	584,222,255,586,443,26,952,226,228,418,518
PTHR19446:SF320	882,201,214,547,618
PTHR19446:SF358	110,809
PTHR19446:SF363	929
PTHR19446:SF352	47,470,261,262,780,265,266,157,498,454,158,
PTHR19446	88,46,274,312,631,159,831,711,953,876,877,9
PTHR19964	398
PTHR20988	41,68
PTHR20988:SF2	40,681
PTHR21311:SF0	692
PTHR21311	691
PTHR22605	951
PTHR22807	212,841,152
PTHR22847	317,182
PTHR22990	299,941,501,568,843,947,131,351,285
PTHR23022	12,761,14,950,16,698,665,667,669,803
PTHR23110	292
PTHR23188	880,797,937
PTHR23227	99,331,640,762,114,126,303,348,128,481
PTHR23227:SF47	115
PTHR23227:SF45	332,304
PTHR23227:SF41	100,641,763,127,129,482
PTHR23259	346,347,927,141
PTHR23278	2
PTHR23504:SF31	288,289,82,72
PTHR23504	290,71,291,83
PTHR23507	723
PTHR23507:SF4	722

PTHR24026	45,37
PTHR24026:SF51	44,371
PTHR24073	220,133,135,765,216,218,98
PTHR24073:SF445	215,217,97
PTHR24073:SF330	134,136
PTHR24073:SF472	764
PTHR24221:SF138	671,28
PTHR24221	907,540,553,776,600,29,733,394,906,296,670
PTHR24230	249
PTHR24260:SF53	231
PTHR24260	375,420,835,230
PTHR24276	311,635
PTHR24291	550,234,105,866,404,391,506,815,240,252
PTHR24292	25,380,383,890,153,267,300,433,785,721,623,
PTHR24305	36,565,898
PTHR24321:SF6	69
PTHR24321	70
PTHR24409	523,316
PTHR24559:SF179	449
PTHR24559	861,643,368,236,448
PTHR24559:SF208	862,644,369
PTHR24559:SF190	235
PTHR28576	66,555,423,801
PTHR28576:SF4	67,556
PTHR31449	895
PTHR31449:SF3	894
PTHR33273	904
PTHR33332	68,353,464,544,247,545,402,546,954,747,748,
PTHR35450	154,474,685,674,686,931,921,800,305,306,95
PTHR19446:SF244	451,275,441,632,720,280,185,87

PTHR19446:SF355	584,222,255,586,443,26,952,226,228,418,518
PTHR19446:SF320	882,201,214,547,618
PTHR19446:SF358	110,809
PTHR19446:SF363	929
PTHR19446:SF352	47,470,261,262,780,265,266,157,498,454,158,
PTHR19446	88,46,274,312,631,159,831,711,953,876,877,9
PTHR19964	398
PTHR20988	41,68
PTHR20988:SF2	40,681
PTHR21311:SF0	692
PTHR21311	691
PTHR22605	951
PTHR22807	212,841,152
PTHR22847	317,182
PTHR22990	299,941,501,568,843,947,131,351,285
PTHR23022	12,761,14,950,16,698,665,667,669,803
PTHR23110	292
PTHR23188	880,797,937
PTHR23227	99,331,640,762,114,126,303,348,128,481
PTHR23227:SF47	115
PTHR23227:SF45	332,304
PTHR23227:SF41	100,641,763,127,129,482
PTHR23259	346,347,927,141
PTHR23278	2
PTHR23504:SF31	288,289,82,72
PTHR23504	290,71,291,83
PTHR23507	723
PTHR23507:SF4	722
PTHR24026	45,37
PTHR24026:SF51	44,371

PTHR24073	220,133,135,765,216,218,98
PTHR24073:SF445	215,217,97
PTHR24073:SF330	134,136
PTHR24073:SF472	764
PTHR24221:SF138	671,28
PTHR24221	907,540,553,776,600,29,733,394,906,296,670
PTHR24230	249
PTHR24260:SF53	231
PTHR24260	375,420,835,230
PTHR24276	311,635
PTHR24291	550,234,105,866,404,391,506,815,240,252
PTHR24292	25,380,383,890,153,267,300,433,785,721,623,
PTHR24305	36,565,898
PTHR24321:SF6	69
PTHR24321	70
PTHR24409	523,316
PTHR24559:SF179	449
PTHR24559	861,643,368,236,448
PTHR24559:SF208	862,644,369
PTHR24559:SF190	235
PTHR28576	66,555,423,801
PTHR28576:SF4	67,556
PTHR31449	895
PTHR31449:SF3	894
PTHR33273	904
PTHR33332	68,353,464,544,247,545,402,546,954,747,748,
PTHR35450	154,474,685,674,686,931,921,800,305,306,95[illegible]

Family name	Genes
PROTEIN YELLOW-RELATED	40
PROTEIN KLO-1-RELATED	117
GLYCOSYL HYDROLASE	588
CUTICLE PROTEIN	379
UNCHARACTERIZED	452
SULFIDE:QUINONE OXIDOREDUCTASE, MITOCHONDRIAL	60
SULFIDE:QUINONE OXIDOREDUCTASE	83
RIBONUCLEASE H1	188
DEPHOSPHO-COA KINASE-RELATED	202
SWI/SNF-RELATED MATRIX-ASSOCIATED ACTIN-DEPENDENT REGULATOR OF CHROMATIN SUBFAMILY-RELATED	2255
ANDROGEN-INDUCED PROTEIN 1-RELATED	80
CONTACTIN-5	94
MALE STERILITY PROTEIN 2-RELATED	227
UNCHARACTERIZED	137
SUBFAMILY NOT NAMED	6
GLUCOSYLCERAMIDASE	114
SUCCINYL-COA SYNTHETASE-RELATED	145
SUCCINYL-COA LIGASE [ADP/GDP-FORMING] SUBUNIT ALPHA, MITOCHONDRIAL	111
EJACULATORY BULB-SPECIFIC PROTEIN 3-RELATED	8
CHEMOSENSORY PROTEIN-RELATED	15
MIP15436P	2
CARBOXYLESTERASE	1013
LIPASE	319
SODIUM/CHLORIDE DEPENDENT TRANSPORTER	740
ADENYLATE AND GUANYLATE CYCLASES	965
GLUCOSYL/GLUCURONOSYL TRANSFERASES	2370
RUNT RELATED	79
STRUCTURAL CONTITUENT OF CUTICLE	300
CUTICULAR PROTEIN 30B-RELATED	8

CUTICULAR PROTEIN 62BC, ISOFORM A	2
CUTICULAR PROTEIN 23B	8
BICOID-INTERACTING PROTEIN RELATED	78
7SK SNRNA METHYLPHOSPHATE CAPPING ENZYME	52
CYSTEINE PROTEASE FAMILY C1-RELATED	1355
LIPRIN-ALPHA, ISOFORM E	16
LAR INTERACTING PROTEIN LIP -RELATED PROTEIN	154
TESTIS-SPECIFIC PROTEIN PBS13 T-COMPLEX 11	125
FATTY ACID HYDROXYLASE	73
FATTY ACID 2-HYDROXYLASE	61
NGG1 INTERACTING FACTOR 3	81
GTP CYCLOHYDROLASE 1 TYPE 2 NIF3L1-RELATED	76
VHS DOMAIN CONTAINING PROTEIN FAMILY	436
GRB10 INTERACTING GYF PROTEIN	139
PERQ AMINO ACID-RICH WITH GYF DOMAIN-CONTAINING PROTEIN 2	19
RIB43A-RELATED	56
DFG10 PROTEIN	61
AN1-TYPE ZINC FINGER PROTEIN 1	57
ARSENITE INDUCUBLE RNA ASSOCIATED PROTEIN AIP-1-RELATED	137
FAMILY NOT NAMED	30
UPF0544 PROTEIN C5ORF45	30
PROTEIN SAEG-1, ISOFORM A	10
REST COREPRESSOR COREST PROTEIN-RELATED	150
FIDIPIDINE	50
COILED-COIL DOMAIN-CONTAINING PROTEIN 93	46
WD40 DOMAIN-CONTAINING PROTEIN	57
IONOTROPIC GLUTAMATE RECEPTOR	1019
IONOTROPIC RECEPTOR 67C-RELATED	7
SUBFAMILY NOT NAMED	14
SUBFAMILY NOT NAMED	20

SUBFAMILY NOT NAMED	11
SUBFAMILY NOT NAMED	43
SUBFAMILY NOT NAMED	3
SUBFAMILY NOT NAMED	23
SUBFAMILY NOT NAMED	27
SUBFAMILY NOT NAMED	3
SUBFAMILY NOT NAMED	107
REVERSE TRANSCRIPTASES	947
MULTIPLE PDZ DOMAIN PROTEIN	210
UNCHARACTERIZED	26
TRANSMEMBRANE PROTEIN 183A-RELATED	26
CONSERVED OLIGOMERIC GOLGI COMPLEX SUBUNIT 8	68
CONSERVED OLIGOMERIC GOLGI COMPLEX COMPONENT 8	68
UNCHARACTERIZED	86
NOP2 YEAST -RELATED NOL1/NOP2/FMU SUN DOMAIN-CONTAINING	274
WD40 REPEAT PROTEIN	1147
F-BOX ONLY PROTEIN	127
TRANSPOSABLE ELEMENT-RELATED	209
BTB DOMAIN TRANSCRIPTION FACTOR	91
FAMILY NOT NAMED	66
BUCENTAUR RELATED	224
SUBFAMILY NOT NAMED	35
SUBFAMILY NOT NAMED	32
SUBFAMILY NOT NAMED	9
RIDDLE	171
SIDESTEP PROTEIN	92
MAJOR FACILITATOR SUPERFAMILY DOMAIN-CONTAINING PROTEIN 10	34
FAMILY NOT NAMED	357
FAMILY NOT NAMED	171
SUBFAMILY NOT NAMED	8

FAMILY NOT NAMED	192
PROTOCADHERIN-LIKE WING POLARITY PROTEIN STAN	12
FAMILY NOT NAMED	3173
RAS-RELATED PROTEIN RAB-3C	15
RAS-RELATED PROTEIN RAB-3D	18
RAB3A, MEMBER RAS ONCOGENE FAMILY	2
LP14331P-RELATED	366
FAMILY NOT NAMED	1188
G-PROTEIN COUPLED RECEPTOR	416
PROTEIN TRY-5	15
FAMILY NOT NAMED	289
FAMILY NOT NAMED	198
FAMILY NOT NAMED	685
FAMILY NOT NAMED	235
FAMILY NOT NAMED	311
3-HYDROXYBUTYRATE DEHYDROGENASE TYPE 2	23
FAMILY NOT NAMED	26
FAMILY NOT NAMED	551
SUBFAMILY NOT NAMED	10
FAMILY NOT NAMED	993
KRAB-A DOMAIN-CONTAINING PROTEIN 2	40
SUBFAMILY NOT NAMED	21
FAMILY NOT NAMED	68
SUBFAMILY NOT NAMED	27
FAMILY NOT NAMED	29
UPF0598 PROTEIN C8ORF82	28
FAMILY NOT NAMED	59
FAMILY NOT NAMED	48
FAMILY NOT NAMED	14
SUBFAMILY NOT NAMED	43

SUBFAMILY NOT NAMED	3
SUBFAMILY NOT NAMED	23
SUBFAMILY NOT NAMED	27
SUBFAMILY NOT NAMED	3
SUBFAMILY NOT NAMED	107
REVERSE TRANSCRIPTASES	947
MULTIPLE PDZ DOMAIN PROTEIN	210
UNCHARACTERIZED	26
TRANSMEMBRANE PROTEIN 183A-RELATED	26
CONSERVED OLIGOMERIC GOLGI COMPLEX SUBUNIT 8	68
CONSERVED OLIGOMERIC GOLGI COMPLEX COMPONENT 8	68
UNCHARACTERIZED	86
NOP2 YEAST -RELATED NOL1/NOP2/FMU SUN DOMAIN-CONTAINING	274
WD40 REPEAT PROTEIN	1147
F-BOX ONLY PROTEIN	127
TRANSPOSABLE ELEMENT-RELATED	209
BTB DOMAIN TRANSCRIPTION FACTOR	91
FAMILY NOT NAMED	66
BUCENTAUR RELATED	224
SUBFAMILY NOT NAMED	35
SUBFAMILY NOT NAMED	32
SUBFAMILY NOT NAMED	9
RIDDLE	171
SIDESTEP PROTEIN	92
MAJOR FACILITATOR SUPERFAMILY DOMAIN-CONTAINING PROTEIN 10	34
FAMILY NOT NAMED	357
FAMILY NOT NAMED	171
SUBFAMILY NOT NAMED	8
FAMILY NOT NAMED	192
PROTOCADHERIN-LIKE WING POLARITY PROTEIN STAN	12

FAMILY NOT NAMED	3173
RAS-RELATED PROTEIN RAB-3C	15
RAS-RELATED PROTEIN RAB-3D	18
RAB3A, MEMBER RAS ONCOGENE FAMILY	2
LP14331P-RELATED	366
FAMILY NOT NAMED	1188
G-PROTEIN COUPLED RECEPTOR	416
PROTEIN TRY-5	15
FAMILY NOT NAMED	289
FAMILY NOT NAMED	198
FAMILY NOT NAMED	685
FAMILY NOT NAMED	235
FAMILY NOT NAMED	311
3-HYDROXYBUTYRATE DEHYDROGENASE TYPE 2	23
FAMILY NOT NAMED	26
FAMILY NOT NAMED	551
SUBFAMILY NOT NAMED	10
FAMILY NOT NAMED	993
KRAB-A DOMAIN-CONTAINING PROTEIN 2	40
SUBFAMILY NOT NAMED	21
FAMILY NOT NAMED	68
SUBFAMILY NOT NAMED	27
FAMILY NOT NAMED	29
UPF0598 PROTEIN C8ORF82	28
FAMILY NOT NAMED	59
FAMILY NOT NAMED	48
FAMILY NOT NAMED	14

PANTHER GO-Slim Molecular function	PANTHER GO-Slim Biological Process	PANTHER GO-Slim Cellular Component
structural molecule activity(GO:0005198)		
structural molecule activity(GO:0005198)		

structural molecule activity(GO:0005198)	
	intracellular(GO:0044464)
	intracellular(GO:0044464)
structural constituent of cytoskeleton(GO:0005198)	cytoskeleton(GO:0043226);intracellular(G
RNA binding(GO:0005488)	
RNA binding(GO:0005488)	

receptor activity(GO:0004872)

receptor activity(GO:0004872)

PANTHER Protein Class

structural protein(PC00211)

esterase(PC00121)

structural protein(PC00211)

structural protein(PC00211)

cytoskeletal protein(PC00085)

RNA binding protein(PC00171)
RNA binding protein(PC00171)

G-protein coupled receptor(PC00197)

G-protein coupled receptor(PC00197)
