# Supplementary material for: The comparative landscape of duplications in Heliconius melpomene and Heliconius cydno
Source: Heredity (Edinb). 2016 Dec 7;118(1):78–87. doi: 10.1038/hdy.2016.107 (PMC5176112; doi:10.1038/hdy.2016.107)
Supplement: Supplementary Table S4 [file hdy2016107x5.pdf]

Chr	Scaffold	Start	End	Size
9	Hmel209007	4344840	4364959	20119
15	Hmel215006	1190144	1196212	6068
18	Hmel218003	221730	429239	207509
21	Hmel221012	779541	796444	16903
2	Hmel202004	1537111	1543050	5939
21	Hmel221001	297653	480497	182844
2	Hmel202006	1791672	1816820	25148
2	Hmel202006	1804029	1835608	31579
2	Hmel202006	4165194	4171714	6520
15	Hmel215047	2849864	2859210	9346
19	Hmel219018	354635	361054	6419

BayeScan log(PO)	BayPass mean XtX	Freq in *H. melpomene*	Freq in *H. cydno*
1.7222	7.95239143	0	0.93
1.8414	8.78515118	0	1
1.894	8.75630075	0	1
1.72	8.35788884	0	0.93
Not Significant	3.97886586	0.65	0.71
Not Significant	3.56709747	0.55	0.5
1.5501	Not Significant	0	0.79
1.0762	Not Significant	0.05	1
0.99213	Not Significant	0	0.93
0.97681	Not Significant	0.05	1
1.2169	Not Significant	0	1

Hmel2 annotation

HMEL009267
intergenic (upstream of *cortex)*
OBP41
HMEL013558
HMEL013559
HMEL003174
HMEL003175
HMEL003862
HMEL003863
HMEL016617
HMEL016621
HMEL016620
intergenic
HMEL011045
HMEL011044
HMEL011042
HMEL011043
HMEL011041
HMEL011040
HMEL011037
HMEL011039
HMEL011038
HMEL015626
HMEL015625
HMEL015624
HMEL012374
HmGr58
HmGr59
HmGr60
HmGr61
HmGr64
HmGr65
