# Supplementary material for: The comparative landscape of duplications in Heliconius melpomene and Heliconius cydno
Source: Heredity (Edinb). 2016 Dec 7;118(1):78–87. doi: 10.1038/hdy.2016.107 (PMC5176112; doi:10.1038/hdy.2016.107)
Supplement: Supplementary Table Legends [file hdy2016107x6.docx]

**Supplementary Table legends**

**Table S1. Illumina paired-end sample information**

Illumina paired-end sequencing information for 20 *H. melpomene* and 14 *H. cydno* butterflies retrieved from public repositories for this study (SRA106228, Kronforst et al. 2013; ERP002440, Martin et al. 2013). *ID*, refers to the code ID given to each sample during this study; *Submission* and *Accession number* are as appear on the public repositories. *Taxon, Sex*, *Country, Latitude, Longitude* and *Sequencing center* are as in the original publications. *Mean* *read-depth*, *Total raw reads*, *Mapped reads* and *Unmapped reads* values calculated after mapping to the *H. melpomene* genome (v2.0) (Davey et al. 2016) with Stampy (v1.0.23; Lunter & Goodson 2011) using default values for all parameters except the substitution rate, which was set to 0.01.

**Table S2. Over-represented PANTHER GO-Slim Biological Processes**

PANTHER over-represented (*P* < 0.05) GO-Slim Biological Processes on the Heliconius Set using the *D. melanogaster* genome as the reference list after Bonferroni correction.

**Table S3. Over-represented PANTHER occurrences without an associated biological process**

PANTHER family name and GO-Slim Molecular function for the over-represented occurences that do not have a GO-Slim Biological Process associated. GO-Slim molecular function and protein class are given when available.

**Table S4. Duplications identified as outliers in the Heliconius Set**

Position, size and summary statistics associated with BayeScan and BayPass. Frequency of the duplication in each species was calculated for all the 12 duplications. Statistics showing the significance of each call are shown for both BayeScan and BayPass. When the duplication event was not significant in one tool *Not significant* was added to the column. Hmel2 annotations are shown for each duplication.
